# Expression of HDACs 1, 3 and 8 Is Upregulated in the Presence of Infiltrating Lymphocytes in Uveal Melanoma

**DOI:** 10.3390/cancers13164146

**Published:** 2021-08-18

**Authors:** Zahra Souri, Aart G. Jochemsen, Annemijn P. A. Wierenga, Wilma G. M. Kroes, Rob M. Verdijk, Pieter A. van der Velden, Gregorius P. M. Luyten, Martine J. Jager

**Affiliations:** 1 Department of Ophthalmology, LUMC, 2333 ZA Leiden, The Netherlands; z.souri@lumc.nl (Z.S.); a.p.a.wierenga@lumc.nl (A.P.A.W.); p.a.van_der_velden@lumc.nl (P.A.v.d.V.); g.p.m.luyten@lumc.nl (G.P.M.L.); 2 Department of Cell and Chemical Biology, LUMC, 2300 RC Leiden, The Netherlands; a.g.jochemsen@lumc.nl; 3 Department of Clinical Genetics, LUMC, 2300 RC Leiden, The Netherlands; w.g.m.kroes@lumc.nl; 4 Department of Pathology, LUMC, 2333 ZA Leiden, The Netherlands; r.m.verdijk@lumc.nl; 5 Department of Pathology, Erasmus MC, 3015 GD Rotterdam, The Netherlands

**Keywords:** uveal melanoma, inflammation, metastasis, chromosome 3, BAP1, histone deacetylase

## Abstract

**Simple Summary:**

Uveal melanoma (UM) is an ocular malignancy which is derived from melanocytes in the uveal tract. Epigenetic regulators such as Histone Deacetylase (HDACs) inhibitors are being tested as treatment of UM metastases. Expression of different HDACs is variable, and some are increased in high-risk tumors with loss of one chromosome 3. As this genetic abnormality is also associated with an inflammatory phenotype, we analyzed whether HDAC expression was influenced by inflammation. In two cohorts of UM cases, expression of several HDACs showed a positive correlation with tumor-infiltrating T cells, while HDACs 2 and 11 showed a negative association with macrophages. Interferon-γ stimulated expression of some HDACs on UM cell lines. These data suggest that cytokines produced by T cells may be responsible for the increased expression of some HDACs in UM with monosomy 3.

**Abstract:**

In Uveal Melanoma (UM), an inflammatory phenotype is strongly associated with the development of metastases and with chromosome 3/BAP1 expression loss. As an increased expression of several Histone Deacetylases (HDACs) was associated with loss of chromosome 3, this suggested that HDAC expression might also be related to inflammation. We analyzed HDAC expression and the presence of leukocytes by mRNA expression in two sets of UM (Leiden and TCGA) and determined the T lymphocyte fraction through ddPCR. Four UM cell lines were treated with IFNγ (50IU, 200IU). Quantitative PCR (qPCR) was used for mRNA measurement of HDACs in cultured cells. In both cohorts (Leiden and TCGA), a positive correlation occurred between expression of HDACs 1, 3 and 8 and the presence of a T-cell infiltrate, while expression of HDACs 2 and 11 was negatively correlated with the presence of tumor-infiltrating macrophages. Stimulation of UM cell lines with IFNγ induced an increase in HDACs 1, 4, 5, 7 and 8 in two out of four UM cell lines. We conclude that the observed positive correlations between HDAC expression and chromosome 3/BAP1 loss may be related to the presence of infiltrating T cells.

## 1. Introduction

Histone Deacetylases (HDACs) are epigenetic enzymes which regulate gene expression primarily by modifying the chromatin structure through the removal of acetyl groups from histones, acting in balance with Histone Acetyl Transferases (HATs). Eighteen types of HDACs have been identified in Homo sapiens, classified into four classes: HDAC Class I includes HDACs 1, 2, 3 and 8, which are located in the nucleus, HDAC Class II includes HDACs 4, 5, 6, 7, 9 and 10, with both nuclear and cytoplasmic locations, while HDAC Class III consists of sirtuins 1–7 and HDAC Class IV is made up by HDAC 11 [1,2]. Several types of HDACs show overexpression in cancer cells, which has been associated with invasive behavior [3,4,5] and a poor clinical outcome, for instance in oral squamous cell carcinoma [6].

Inhibitors of HDACs (HDACi) are being investigated as treatment of a wide range of malignancies such as leukemia [7], and Uveal Melanoma (UM), which constitute a rare ocular tumor arising from the uveal tract (ClinicalTrials.gov NCT-4133048). Primary UM mainly involves the choroid but may also develop in the iris or ciliary body and gives rise to metastases in 50% of cases [8]. Specific somatic mutations and chromosome aberrations in primary UM are associated with the risk of metastases: loss of one chromosome 3 (monosomy 3) and a mutation in the BRCA1-associated protein 1 (BAP1) gene on the other chromosome 3 are associated with a very high risk of developing metastases [9,10]. In a recent study, we described that high-risk UM showed an elevated expression of several HDACs, which was associated with monosomy 3 [11].

Monosomy 3/loss of BAP1 expression is related to an inflammatory phenotype, which is characterized by the presence of tumor-infiltrating lymphocytes (TILs), tumor-associated macrophages (TAMs) and high expression levels of the Major Histocompatibility complex (MHC) proteins HLA Class I and HLA Class II [12,13,14,15,16]. Infiltration of UM with immune cells is associated with an increased risk of metastasis [17,18,19]. Specific genetic abnormalities are associated with the development of inflammation in UM: extra copies of chromosome 8q are related to macrophage influx, while monosomy 3/BAP1 loss is also associated with the presence of lymphocytes [17,20]. As differences in expression levels of HDACs were related to monosomy 3/BAP1 loss, we considered a relation between their expression and inflammation.

We set out to investigate the hypothesis that the increased level of expression of HDACs in some tumors is related to the inflammatory phenotype, due to the production of cytokines produced by infiltrating leukocytes. We analyzed this by determining HDAC expression and the presence of an inflammatory phenotype in two cohorts of UM patients, and by treating four UM cell lines with IFNγ. We selected two BAP1-positive and two BAP1-negative cell lines, of which two were derived from a primary tumor and two from a metastasis.

## 2. Materials and Methods

### 2.1. Study Population

The studied tumors came from 64 eyes that underwent an enucleation for UM at the Leiden University Medical Center (LUMC), Leiden, The Netherlands, between 1999 and 2008. In this group, 51% of the patients were male and 49% female. Their mean age at the time of enucleation was 61 years. The mean follow-up time (defined as the time period between enucleation and either date of last follow-up or death) was 83 months (range 2 to 229 months). Follow-up was updated in 2020. At the end of follow up, 17 patients (27%) were alive, 37 patients (58%) had died because of metastases, four (6%) had died because of other causes, while for six patients (9%) the cause of death was unknown.

MRNA levels of patients included in the TCGA database were investigated for validation of our results (*n* = 80) [19].

This work has been carried out in accordance with the Code of Ethics of the World Medical Association (Declaration of Helsinki). Material was included in the Leiden Ophthalmic Oncology Biobank (B14.003/DH/sh) and approved by the LUMC Biobank committee and the LUMC METC committee (19/10/2016, code G16.076/NV/gk).

### 2.2. Chromosome Analysis

Tumour DNA was isolated using the QIAmp DNA Mini kit (Qiagen, Venlo, The Netherlands). Single-nucleotide polymorphism (SNP) analysis was performed using an Affymetrix 250K_NSP or Affymetrix SNP 6.0 array to detect chromosome 3 loss. Additional copies of chromosome 8q were detected by Affymetrix SNP 6.0 array and analyzed using the GISTIC 2.0 algorithm [20,21].

### 2.3. Tumour Gene Expression

RNA was isolated using the RNeasy mini kit (Qiagen, Venlo, The Netherlands). Gene expression levels were determined using an Illumina HT12v4 array (Illumina, Inc., San Diego, CA, USA) and data were obtained for expression of epigenetic regulators (HDAC1, HDAC2, HDAC3, HDAC4, HDAC6, HDAC7, HDAC8, HDAC9 and HDAC11) and infiltrate markers (CD3E, CD8A and CD68). Information regarding the Illumina probe numbers and gene expression levels has been published [11].

### 2.4. Immunohistochemistry

In order to investigate BAP1 protein expression, tumors were divided into BAP1-positive or -negative based on nuclear staining with a mouse monoclonal antibody against amino acids 430–729 of human BAP1 (clone sc-28383, 1:50 dilution, Santa Cruz Biotechnology, Dallas, TX, USA) as previously described [22].

### 2.5. Droplet Digital PCR (ddPCR)

Droplet digital PCR (ddPCR) was used in order to measure the T cell fraction as previously described [23,24], applying a specifically designed probe directed at a locus of the TCR-β gene.

### 2.6. Cell Lines and Cell Culture

Four UM Cell lines were used in this study: the OMM1 cell line was previously established from a metastasis by Dr G.P.M. Luyten (LUMC, Leiden, The Netherlands) [25]. OMM2.5 was also derived from a metastasis and obtained from Dr B.R. Ksander (Schepens Eye Research Institute, Boston, MA, USA) [26]. Both are BAP1-positive and cultured in Roswell Park Memorial Institute Medium 1640 (RPMI) Dutch modified media (Life Technologies, Europe bv, Bleiswijk, The Netherlands) supplemented with 10% fetal bovine serum (FBS) (Greiner Bio-one, Alphen a/d Rijn, The Netherlands), 1% GlutaMAX and 1% penicillin/streptomycin (Life Technologies).

Two BAP1-negative UM cell lines (MP46 and MP38) derived from primary tumors were provided by the Curie Institute, Paris, France [27] and cultured in Iscove’s modified Dulbecco medium (IMDM) (Life Technologies), supplemented with 20% FBS (Greiner Bio-one) and 1% penicillin/streptomycin (Life Technologies). The cell lines represent both GNA11 (OMM1) and GNAQ mutations (OMM2.5, MP38, MP46).

### 2.7. Quantitative Real-Time Polymerase Chain Reaction (qPCR)

The procedure for RNA isolation and quantitative real-time polymerase chain reaction (qPCR) has been described previously [28]. In summary, total RNA was extracted from cells using an RNeasy Mini Kit (Qiagen Benelux B.V., Venlo, The Netherlands). The IScript cDNA synthesis kit (Bio-Rad Laboratories B.V., Veenendaal, The Netherlands) was useor complementary cDNA synthesis according to the manufacturer’s instructions. Quantitative real-time polymerase chain reaction (qPCR) was performed in three independent experiments using the CFX-384 machine (Bio-Rad), with triplicates. Data were analyzed using CFX manager 3.1 (Bio-Rad). CT values of genes of interest were normalized against the geometric mean of housekeeping genes *RPS11* and *CAPNS1*. The sequences of primers used in the study are shown in Table 1.

### 2.8. Statistics

Data were analyzed with SPSS software version 22.0 (SPSS, nc., Chicago, IL, USA). Spearman correlation was performed in order to test correlations between non-parametric data. Pearson’s chi square test was used for categorical data analysis. Graphs were obtained using GraphPad Prism version 5.0 for Windows (GraphPad Software, La Jolla, CA, USA). An Independent t test was used to compare qPCR data.

## 3. Results

### 3.1. HDAC Expression Is Related to Clinical and Genetic Tumour Characteristics

**Figure 1:**
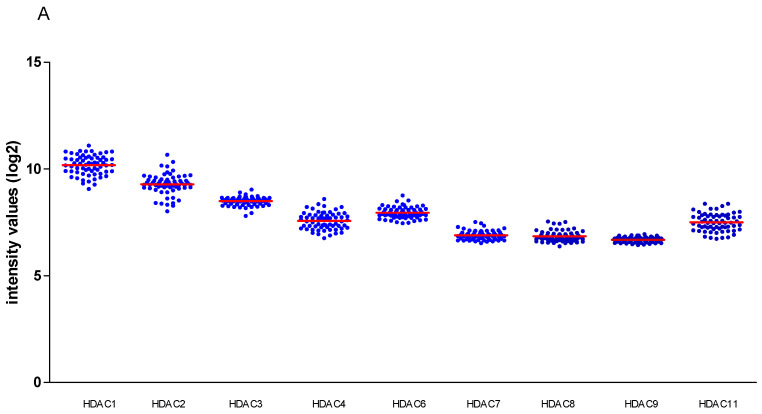

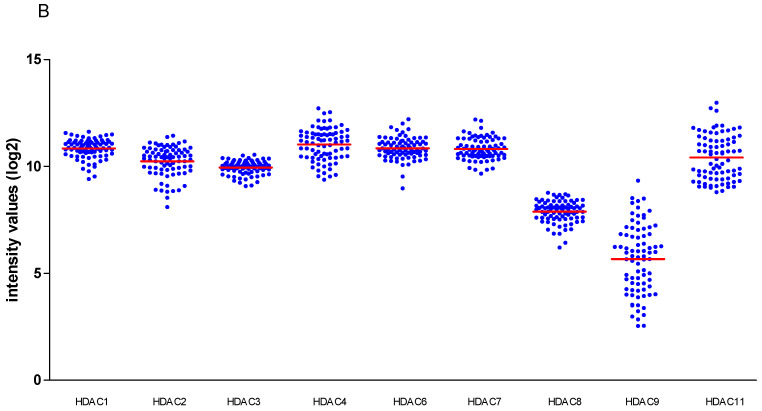
Distribution of HDAC mRNA in (**A**) 64 UM from Leiden; (**B**) 80 UM from the TCGA cohort. Horizontal bars indicate mean gene expression.

When we looked at the mRNA levels of the different HDACs, we noticed a moderate variable expression in the Leiden cohort (Figure 1A) as well as in the TCGA panel (Figure 1B). We already knew that expression of some of the HDACs was related to the tumour’s chromosome 3 status, but now also investigated the possible association between HDACs and patient (age and gender) and tumor characteristics, such as cell type, tumor location (involvement of the ciliary body), and tumor size (indicated as cTNM stage) in the Leiden cohort of 64 cases as well as in the TCGA cohort (mRNA expression) of 80 cases. BAP1 expression was determined by immunohistochemical staining in 55 cases of the Leiden cohort.

When we looked at the parameters age and gender, we did not find any significant associations with the level of HDAC mRNA expression. Increased expression of HDACs 1 and 8 was associated with the presence of epithelioid cells (*p* = 0.002 and *p* = 0.005), increased HDAC4 expression was associated with ciliary body involvement (*p* = 0.04, data not shown) and high cTNM stage (*p* = 0.04). Expression of HDACs 1, 4, and 8 was higher in tumors with monosomy 3 (*p* = 0.002, *p* = 0.01, and *p* < 0.001, respectively), while HDAC11 showed a lower expression in cases with monosomy 3 (*p* < 0.001). Increased expression of HDAC4 and HDAC8 was associated with loss of BAP1 staining (*p* = 0.004, *p* = 0.001, respectively), while the opposite relation was observed for HDAC11 (*p* = 0.004) (Table 2).

As expected, when we looked at the TCGA cohort, HDACs 4 and 8 but also HDAC3 showed a negative correlation with the BAP1 mRNA expression level (*p* < 0.001, *p* < 0.001, *p* = 0.02), while HDACs 6 and 11 (*p* < 0.001, *p* < 0.001) showed a positive correlation (Supplementary Table S1).

### 3.2. HDACs and Relation with Infiltrating Leukocytes

As already observed [11] and confirmed (Table 2), expression levels of some of the HDACs were related to the tumor chromosome 3/BAP1 status [11]. As monosomy 3 is associated with the presence of an inflammatory phenotype, we speculated that the epigenetic enzymes might be upregulated due to the presence of infiltrating leukocytes which produce cytokines that stimulate HDAC expression. We set out to test this hypothesis (Table 3).

Expression of HDACs 1, 3, 7 and 8 was positively correlated with the expression of the T cell markers CD3E and CD8A, while HDAC11 showed negative correlations with these markers. HDAC2 and HDAC11 were inversely correlated with macrophage marker CD68 (*p* < 0.001, *p* = 0.001).

When performing the same analysis using the TCGA cohort, the results showed that HDACs 1, 3 and 8 were (again) positively correlated with to the presence of CD3E and CD8A TILs, while HDACs 2 and 11 were (again) negatively correlated with CD3E (*p* = 0.003, *p* = 0.002), and HDAC2 and HDAC9 with CD68 (*p* < 0.001, *p* < 0.001) (Table 4).

**Figure 2:**
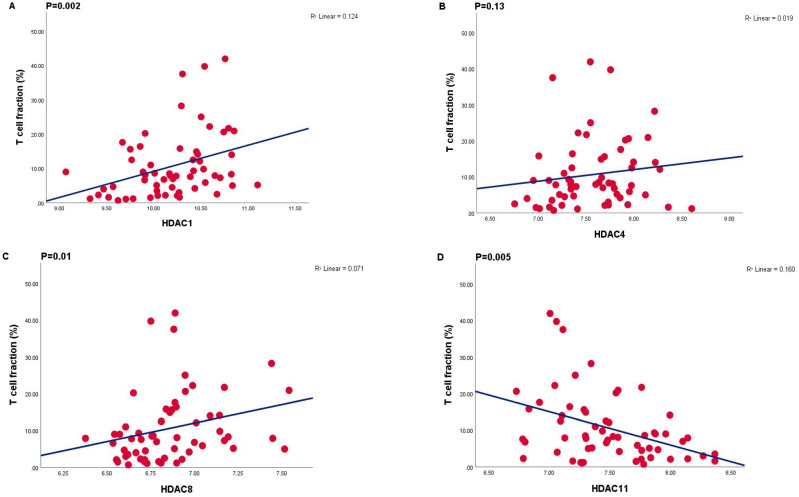
Correlations between mRNA expression levels (determined by Illumina array) of different HDACs and T cell fraction (%) (Determined by ddPCR) (*n* = 59). (**A**) HDAC1 R2 = 0.124, *p* = 0.002, (**B**) HDAC4 R2 = 0.019, *p* = 0.13, (**C**) HDAC8 R2 = 0.071, *p* = 0.01, (**D**) HDAC11 R2 = 0.160, *p* = 0.005.

In order to obtain the percentage of T cells as part of the tumor as an absolute count, we used a ddPCR technique to quantify the T cell infiltrate (Supplementary Table S2). A comparison of the T cell fraction versus the expression of HDACs 1, 4, 8 and 11 is provided in Figure 2. Significant positive correlations were observed between T cell fraction and HDACs 1, 3 and 8, and a negative correlation with HDACs 6 and 11.

### 3.3. HDAC Expression in UM Cell Lines

**Figure 3:**
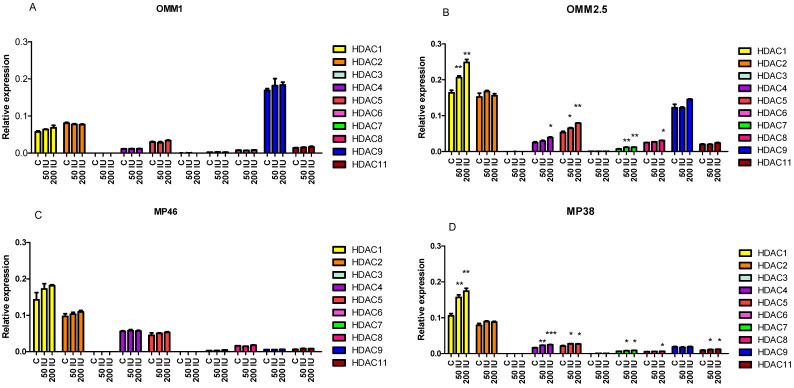
HDAC mRNA levels after 48 h of treatment with IFNγ compared to control. MRNA expression was determined by qPCR in four UM cell lines (**A–D**). C: control; 50IU: 50IU IFNγ; 200IU: 200IU IFNγ. Using an Independent *t*-test, * *p* ≤ 0.05, ** *p* < 0.01, *** *p* < 0.001. Error bars indicate the standard error of the mean.

The correlation between some infiltrate markers and expression levels of HDACs 1, 3, 4, 6 and 8 could suggest that their expression levels are influenced by the presence of infiltrating cells; this may be due to the production of cytokines such as interferon-γ (IFNγ). To test this option, we looked at the level of expression of a range of HDACs in four UM cell lines before and after exposing them to two different doses of IFNγ for 48 h (Figure 3). HLA-A and HLA-B mRNA levels were measured as positive controls and showed a strong increase after IFNγ exposure (Supplementary Figure S1).

We observed low levels of HDACs 3, 6 and 7 in all cell lines, with slightly higher levels of HDACs 8 and 11. IFNγ induced a slight but significant increase in HDACs1, 4, 5, 7 and 8 in cell lines OMM2.5 and MP38 cell lines, and of HDAC11 in MP38. No significant changes were observed in cell lines OMM1 and MP46.

## 4. Discussion

Previously, we reported that expression of several HDACs was increased in high-risk UM with monosomy 3/loss of BAP1 and gain of 8q [11]. However, we observed variation in expression of several HDACs. As we wanted to get a better insight into the cause of this variable expression, we determined whether expression was related to any specific histological or genetic tumor characteristics. For this, we first used the set of 64 primary UM from Leiden which had been analyzed for chromosome 3 status, BAP1 immunohistochemical staining and mRNA expression levels. Expression levels of two HDACs (1 and 8) were higher in case of epithelioid cells, and three HDACs (1, 4, 8) were increased in tumors with monosomy 3, an indicator of bad prognosis in this disease; as monosomy 3 is related to the presence of an inflammatory phenotype with increased levels of HLA Class I expression and the presence of TIL and TAM, we considered the option that the inflammatory microenvironment might be responsible for the upregulation of HDACs in UM. In the Leiden cohort, we observed that four of the HDACs (HDACs 1, 3, 7 and 8) showed a positive correlation between expression levels and TIL, while this was the case for three of the HDACs in the TCGA study (HDACs 1, 3 and 8). HDAC11 showed a consistently negative association with TIL as well as TAM.

In order to test our hypothesis that the presence of infiltrating leukocytes led to HDAC expression through the production of cytokines, we treated four UM cell lines (two BAP1 positive ones: OMM1 and OMM2.5, and two BAP1-negative ones: MP46 and MP38) with two different doses of IFNγ, an inflammatory cytokine normally produced by immune cells. After 48 h incubation with IFNγ, a slight induction of HDAC expression was seen in two of the four cell lines (OMM2.5 and MP38). This suggests a potential difference in the regulation of HDACs between individuals in response to IFNγ, although in this small group, the sensitivity could not be related to differences in tumor genetics.

HDACs contribute towards malignancy: they block the activity of cell cycle inhibitors, inhibit differentiation and apoptosis and thereby enhance uncontrolled proliferation and survival of cancer cells. HDAC expression has been associated with the invasive and stem cell behavior of UM cells: a change in epigenetic regulation has been proposed by Landreville in 2012, who noticed that loss of melanocytic behavior and a shift toward stem cell behavior occurred during BAP1 inactivation [29]; this could be relevant to the increase in several HDACs after chromosome 3/BAP1 loss. However, we did not notice a basic difference in expression between the BAP1-positive and BAP1-negative cell lines.

Besides their role in the induction of malignancy and invasive behavior, HDACs are also involved in inflammatory processes, as HDACs could act as inducers of interferon-stimulated genes: inhibition of HDACs by trichostatin inhibited the recruitment of RNA poly II and expression of the ISRE element-containing genes ISG54, ISG15 and ISG56 in primary human fibroblasts. This suggests that HDACs are necessary for the expression of such genes and may regulate inflammatory processes [30]. This may constitute a positive feedback loop, in which inflammation-related cytokines stimulate expression of some HDACs, which subsequently stimulate inflammatory cellular pathways. As we saw a negative association between HDAC2 and the presence of macrophages, some HDACs may have a negative immunomodulatory effect.

We previously reported that HDACs are associated with HLA expression, which is part of the inflammatory phenotype in UM: mRNA expression of HDACs (1, 4 and 8) was positively associated with HLA-A and HLA-B expression [11]. When we now look at the relations with lymphocyte markers, HDACs 1, 3, 7 and 8 show the most consistent positive association, and HDACs 2 and 11 a strong negative association.

HDAC1 is involved in the expression of type I IFN-responding genes: when cells were treated with the HDAC inhibitor Sodium Butyrate, expression of IFN-stimulated genes was blocked in several human cell lines; depletion of HDAC1 by siRNA reduced the mRNA expression level of ISG54 [31].

HDAC8 has been shown to have increased enzymatic activity and play a pathogenic role in pulmonary asthma; when mice in a model of allergic asthma were exposed to ovalbumin (OVA), the level of HDAC8 protein expression was significantly increased in the lungs, together with high numbers of CD68 and CD163 macrophages. Treatment with the specific HDAC8 inhibitor PCI-34051 reduced these effects [32]. Another study found that PCI-34051 downregulated inflammatory cytokines in peripheral blood mononuclear cells (IL-18, IL-1b, MIP-1b, MCP-1, TNFa and IL-6) [33]. These reports demonstrate a role for HDAC8 in the induction of inflammation.

HDAC2 and HDAC11 showed a negative correlation with markers of inflammation such as the presence of lymphocytes and macrophages, both in the Leiden cohort as well as the TCGA cohort. In a study on human cervical cancer cell lines, HDAC2 was found to inhibit transcription of the genes of the Major Histocompatibility Complex, which are associated with the inflammatory phenotype in UM [34]. HDAC2 has been studied extensively in relation to pulmonary inflammation: HDAC2 mRNA and protein expression were reduced in lung epithelial cells and macrophages after exposure to hypoxia [35]. We have previously shown that high-risk UM are characterized by a hypoxic environment [36].

HDAC11 has a low expression in high-risk UM [11,37]. The best explanation for this phenomenon is that HDAC11 is located on chromosome 3 and expression is decreased after loss of one of the two chromosomes 3. We previously published that monosomy 3 is associated with tumor inflammation: here, we observe a negative correlation between HDAC11 expression and inflammatory TIL and TAM markers. However, a low HDAC11 level may still contribute to the invasiveness of malignant cells: low levels of this HDAC have been reported to increase the risk of metastasis in breast cancer [38].

The expression of some of the HDACs not only shows a correlation with infiltrating lymphocytes, but has also been shown to associate with immune checkpoint expression, suggesting a possible role for these HDACs in the immune evasion of tumor cells: high levels of HDACs 1, 3, 6 and 8 were positively correlated with expression of the B7 homolog 1 checkpoint inhibitor (B7-H1) in gastric cancer. When gastric cell lines were treated with the HDAC inhibitor vorinostat after IFNγ induction, B7-H1 was reduced, showing that HDACs play a role in the IFNγ enhancement of B7-H1 in gastric cancer and are involved in the evasion phenotype of these malignant cells [39]. When previously investigated the effect of the pan-HDAC inhibitor Quisinostat on cultured UM cells to see how it might impact HLA; due to its function as a chromatin unwounding factor, Quisinostat induced HLA-A and -B expression in UM cell lines [11]. More work is needed to investigate how HDAC inhibitors influence the balance between inhibiting and stimulating immune responses.

## 5. Conclusions

We report a positive correlation between expression of HDACs 1, 3 and 8 and the presence of a T-cell infiltrate in two cohorts of enucleated UM, while a negative correlation was observed between HDACs 2 and 11 and infiltrating macrophages. After stimulation of UM cell lines with IFNγ, a slight increase in HDACs 1, 4, 5, 7 and 8 occurred in two out of four UM cell lines. These data indicate that inflammatory cytokines produced by infiltrating immune cells may stimulate upregulation of HDACs in UM, thereby contributing to the malignant behavior in this disease.

## Figures and Tables

**Table 1:** Sequences of primers used in the qPCR test.

Primers	Forward	Reverse
HDAC1	5′-CATCGCTGTGAATTGGGCTG	5′-CCCTCTGGTGATACTTTAGCAGT
HDAC2	5′-CATGGCGTACAGTCAAGGAG	5′-ATAATTTCCAATATCACCGTCGTAG
HDAC3	5′-AGTTCTGCTCGCGTTACACA	5′-CCGAGGGTGGTACCTCAAAC
HDAC4	5′-TGGGAGTTTGGAGCTCGTTG	5′-AGTCCATCTGGATGGCTTTGGG
HDAC5	5′-TGGTCTACGACACGTTCATGCT	5′-TCAGGGTGCACGTGTGTGTT
HDAC6	5′-GGAGAATCAGATCGCAACCGC	5′-ACTGGGGGTTCTGCCTACTT
HDAC7	5′-GACAAGAGCAAGCGAAGTGC	5′-GAGGTGTGGGGACACTGTAG
HDAC8	5′-CCAAGAGGGCGATGATGATC	5′-GTGGCTGGGCAGTCATAACC
HDAC9	5′-GAGGACGAGAAAGGGCAGTG	5′-GTACCAGAGCTTGGGATGGC
HDAC11	5′-TGTCTACAACCGCCACATCT	5′-GGTGCCTGCATTGTATACC
RPS11	5′-AAGCAGCCGACCATCTTTCA	5′-CGGGAGCTTCTCCTTGCC
CAPNS1	5′-ATGGTTTTGGCATTGACACATG	5′-GCTTGCCTGTGGTGTCGC

**Table 2:** Clinico-pathological characteristics according to low and high HDAC expression. Groups were separated according to the median into low (L) expression and high expression (H). Using Pearson’s Chi Square test, *p* ≤ 0.05 is considered significant (indicated in bold). Numbers in brackets represent percentages. IHC = Immunohistochemical.

Characteristics	HDAC1			HDAC2			HDAC3			HDAC4			HDAC6			HDAC7			HDAC8			HDAC11		
	L	H	*P*	L	H	*P*	L	H	*P*	L	H	*P*	L	H	*P*	L	H	*P*	L	H	*P*	L	H	*P*
**Age (Years) at Enucleation (*n* = 64)**																								
≤60	17 (26)	14 (22)		13 (20)	18 (28)		18 (28)	13 (20)		19 (30)	12 (19)		14 (22)	17 (26)		15 (23)	16 (25)		17 (26)	14 (22)		12 (19)	19 (30)	
>60	15 (23)	18 (28)	0.45	17 (26)	16 (25)	0.44	13 (20)	20 (31)	0.13	13 (20)	20 (31)	0.08	18 (28)	15 (23)	0.45	18 (28)	15 (23)	0.62	17 (26)	16 (25)	0.8	19 (30)	14 (22)	0.13
**Gender (*n* = 64)**																								
Male	15 (23)	18 (28)		18 (28)	15 (23)		18 (28)	15 (23)		16 (25)	17 (26)		17 (26)	16 (25)		15 (23)	18 (28)		17 (26)	16 (25)		15 (23)	18 (28)	
Female	17 (26)	14 (22)	0.45	12 (19)	19 (30)	0.2	13 (20)	18 (28)	0.31	16 (25)	15 (23)	0.8	15 (23)	16 (25)	0.8	18 (28)	13 (20)	0.31	17 (26)	14 (22)	0.8	16 (25)	15 (23)	0.62
**Cell Type (*n* = 64)**																								
Spindle	17 (27)	5 (8)		9 (14)	13 (20)		14 (22)	8 (12)		13 (20)	9 (14)		9 (14)	13 (20)		12 (19)	10 (16)		17 (27)	5 (8)		9 (14)	13 (20)	
Mixed/epithelioid	15 (23)	27 (42)	**0.002**	21 (33)	21 (33)	0.49	17 (27)	25 (39)	0.08	19 (30)	23 (36)	0.3	23 (36)	19 (30)	0.3	21 (33)	21 (33)	0.73	17 (27)	25 (39)	**0.005**	22 (34)	20 (31)	0.38
**cTNM Stage (*n* = 62)**																								
cTNM Stage I-IIB	19 (31)	18 (29)		17 (27)	20 (32)		16 (26)	21 (34)		23 (37)	14 (23)		20 (32)	17 (27)		22 (35)	15 (24)		20 (32)	17 (27)		16 (26)	21 (34)	
cTNM Stage IIIA-IIIC	11 (18)	14 (22)	0.57	13 (21)	12 (19)	0.64	13 (21)	12 (19)	0.5	9 (14)	16 (26)	**0.04**	11 (18)	14 (23)	0.44	9 (14)	16 (26)	0.07	13 (21)	12 (19)	0.87	13 (21)	12 (19)	0.5
**Chromosome 3 Status (*n* = 64)**																								
Disomy 3	18 (28)	6 (9)		13 (20)	11 (17)		15 (23)	9 (14)		17 (26)	7 (11)		10 (16)	14 (22)		16 (25)	8 (12)		21 (33)	3 (5)		4 (6)	20 (31)	
Monosomy 3	14 (22)	26 (41)	**0.002**	17 (27)	23 (36)	0.36	16 (25)	24 (37)	0.08	15 (23)	25 (40)	**0.01**	22 (34)	18 (28)	0.3	17 (26)	23 (36)	0.06	13 (20)	27 (42)	**<0.001**	27 (42)	13 (20)	**<0.001**
**BAP1 IHC staining (*n* = 55)**																								
BAP1-positive	16 (29)	9 (16)		11 (20)	14 (25)		15 (27)	10 (18)		18 (33)	7 (13)		14 (25)	11 (20)		18 (33)	7 (13)	0.06	20 (36)	5 (9)		7 (13)	18 (33)	
BAP1-negative	12 (22)	18 (33)	0.08	13 (24)	17 (31)	0.96	11 (20)	19 (34)	0.08	10 (18)	20 (36)	**0.004**	16 (29)	14 (25)	0.84	14 (25)	16 (29)		10 (18)	20 (36)	**0.001**	20 (36)	10 (18)	**0.004**

**Table 3:** Correlation between mRNA expression levels (determined by Illumina array) of different HDACs and expression of TILs (CD3E and CD8A) and TAMs (CD68) in the LUMC cohort (*n* = 64). R = two-tailed Spearman correlation coefficient. *p* ≤ 0.05 is considered significant (indicated in bold).

	CD3E		CD8A		CD68	
	R	*P*	R	*P*	R	*P*
**HDAC1**	0.340	**0.006**	0.407	**0.001**	0.082	0.52
**HDAC2**	−0.197	0.12	−0.153	0.23	−0.430	**<0.001**
**HDAC3**	0.256	**0.04**	0.315	**0.01**	0.129	0.31
**HDAC4**	0.074	0.56	0.217	0.08	0.029	0.82
**HDAC6**	−0.109	0.39	−0.228	0.07	0.017	0.89
**HDAC7**	0.329	**0.01**	0.395	**0.001**	0.260	**0.04**
**HDAC8**	0.350	**0.005**	0.429	**<0.001**	0.241	**0.05**
**HDAC9**	−0.136	0.28	−0.168	0.18	−0.034	0.79
**HDAC11**	−0.259	**0.04**	−0.254	**0.04**	−0.415	**0.001**

**Table 4:** Correlation between mRNA expression levels of different HDACs and expression of TILs (CD3E and CD8A) and TAMs (CD68) in the TCGA cohort (*n* = 80). R = two-tailed Spearman correlation coefficient. *p* ≤ 0.05 is considered significant (indicated in bold).

	CD3E		CD8A		CD68	
	R	*P*	R	*P*	R	*P*
**HDAC1**	0.409	**<0.001**	0.446	**<0.001**	−0.124	0.27
**HDAC2**	−0.329	**0.003**	−0.143	0.20	−0.445	**<0.001**
**HDAC3**	0.323	**0.003**	0.373	**0.001**	0.201	0.07
**HDAC4**	0.076	0.50	0.229	**0.04**	0.076	0.50
**HDAC6**	−0.211	0.06	−0.270	**0.01**	0.074	0.51
**HDAC7**	0.003	0.98	−0.089	0.43	0.060	0.59
**HDAC8**	0.364	**0.001**	0.478	**<0.001**	−0.202	0.07
**HDAC9**	−0.098	0.39	0.020	0.86	−0.396	**<0.001**
**HDAC11**	−0.339	**0.002**	−0.452	**<0.001**	−0.169	0.13

## Data Availability

Data from the Leiden cohort are accessible through GEO Series accession number GSE84976 (https://www.ncbi.nlm.nih.gov/geo/query/acc.cgi?acc=GSE84976).

## Supplementary Materials

The following are available online at https://www.mdpi.com/article/10.3390/cancers13164146/s1, Table S1: Correlation between mRNA expression levels (determined by Illumina array) of different HDACs and expression of BAP1 in the TCGA cohort (*n* = 80). R = two-tailed Spearman correlation coefficient. *p* ≤ 0.05 is considered significant (indicated in bold). Table S2: Correlation between mRNA expression levels (determined by Illumina array) of different HDACs and T cell fraction (%) as determined by ddPCR (*n* = 59). R = two-tailed Spearman correlation coefficient. *p* ≤ 0.05 is considered significant (indicated in bold). Figure S1: Influence of adding IFNγ on HLA-A and HLA-B mRNA expression on cultured UM cell lines.

## References

[1] Moschos M.M., Dettoraki M., Androudi S., Kalogeropoulos D., Lavaris A., Garmpis N., Damaskos C., Garmpi A., Tsatsos M. (2018). The Role of Histone Deacetylase Inhibitors in Uveal Melanoma: Current Evidence. Anticancer Res..

[2] De Ruijter A.J., Van Gennip A.H., Caron H.N., Kemp S., Van Kuilenburg A.B. (2003). Histone deacetylases (HDACs): Characterization of the classical HDAC family. Biochem. J..

[3] Fritzsche F.R., Weichert W., Röske A., Gekeler V., Beckers T., Stephan C., Jung K., Scholman K., Denkert C., Dietel M. (2008). Class I histone deacetylases 1, 2 and 3 are highly expressed in renal cell cancer. BMC Cancer.

[4] Niegisch G., Knievel J., Koch A., Hader C., Fischer U., Albers P., Schulz W. (2013). Changes in histone deacetylase (HDAC) expression patterns and activity of HDAC inhibitors in urothelial cancers. Urol. Oncol. Semin. Orig. Investig..

[5] Yang H., Salz T., Zajac-Kaye M., Liao D., Huang S., Qiu Y. (2014). Overexpression of histone deacetylases in cancer cells is controlled by interplay of transcription factors and epigenetic modulators. FASEB J..

[6] Chang H.-H., Chiang C.-P., Hung H.-C., Lin C.-Y., Deng Y., Kuo M.Y.-P. (2009). Histone deacetylase 2 expression predicts poorer prognosis in oral cancer patients. Oral Oncol..

[7] Venugopal B., Baird R., Kristeleit R.S., Plummer R., Cowan R., Stewart A., Fourneau N., Hellemans P., A Elsayed Y., McClue S. (2013). A Phase I Study of Quisinostat (JNJ-26481585), an Oral Hydroxamate Histone Deacetylase Inhibitor with Evidence of Target Modulation and Antitumor Activity, in Patients with Advanced Solid Tumors. Clin. Cancer Res..

[8] Kivela T., Simpson E.R., Grossniklaus H.E., Jager M.J., Singh A.D., Caminal J.M., Pavlick A.C., Kujala E., Coupland S.E., Finger P. (2017). Uveal melanoma. AJCC Cancer Staging Manual.

[9] Harbour J.W., Onken M.D., Roberson E.D.O., Duan S., Cao L., Worley L.A., Council M.L., Matatall K.A., Helms C., Bowcock A.M. (2010). Frequent Mutation of BAP1 in Metastasizing Uveal Melanomas. Science.

[10] Smit K.N., Van Poppelen N.M., Vaarwater J., Verdijk R., Van Marion R., Kalirai H., E Coupland S., Thornton S., Farquhar N., Dubbink H.-J. (2018). Combined mutation and copy-number variation detection by targeted next-generation sequencing in uveal melanoma. Mod. Pathol..

[11] Souri Z., Jochemsen A.G., Versluis M., Wierenga A.P., Nemati F., Van Der Velden P.A., Kroes W.G., Verdijk R.M., Luyten G.P., Jager M.J. (2020). HDAC Inhibition Increases HLA Class I Expression in Uveal Melanoma. Cancers.

[12] Durie F.H., Campbell A.M., Lee W.R., Damato B.E. (1990). Analysis of lymphocytic infiltration in uveal melanoma. Investig. Ophthalmol. Vis. Sci..

[13] de Waard-Siebinga I., Hilders C.G.J.M., Hansen B.E., van Delft J.L., Jager M.J. (1996). HLA expression and tumor-infiltrating immune cells in uveal melanoma. Graefe’s Arch. Clin. Exp. Ophthalmol..

[14] Blom D.J., Luyten G.P., Mooy C., Kerkvliet S., Zwinderman A.H., Jager M.J. (1997). Human leukocyte antigen class I expression. Marker of poor prognosis in uveal melanoma. Investig. Ophthalmol. Vis. Sci..

[15] Ericsson C., Seregard S., Bartolazzi A., Levitskaya E., Ferrone S., Kiessling R., Larsson O. (2001). Association of HLA class I and class II antigen expression and mortality in uveal melanoma. Investig. Ophthalmol. Vis. Sci..

[16] Souri Z., Wierenga A.P., Mulder A., Jochemsen A.G., Jager M.J. (2019). HLA Expression in Uveal Melanoma: An Indicator of Malignancy and a Modifiable Immunological Target. Cancers.

[17] Maat W., Ly L.V., Jordanova E.S., De Wolff-Rouendaal D., Schalij-Delfos N.E., Jager M.J. (2008). Monosomy of Chromosome 3 and an Inflammatory Phenotype Occur Together in Uveal Melanoma. Investig. Opthalmol. Vis. Sci..

[18] Bronkhorst I.H.G., Vu T.H.K., Jordanova E.S., Luyten G.P.M., Burg S.H.V.D., Jager M.J. (2012). Different Subsets of Tumor-Infiltrating Lymphocytes Correlate with Macrophage Influx and Monosomy 3 in Uveal Melanoma. Investig. Opthalmol. Vis. Sci..

[19] Robertson A.G., Shih J., Yau C., Gibb E.A., Oba J., Mungall K.L., Hess J.M., Uzunangelov V., Walter V., Danilova L. (2017). Integrative Analysis Identifies Four Molecular and Clinical Subsets in Uveal Melanoma. Cancer Cell.

[20] Gezgin G., Dogrusöz M., Van Essen T.H., Kroes W.G.M., Luyten G.P.M., Van Der Velden P.A., Walter V., Verdijk R.M., van Hall T., Van Der Burg S.H. (2017). Genetic evolution of uveal melanoma guides the development of an inflammatory microenvironment. Cancer Immunol. Immunother..

[21] Versluis M., de Lange M.J., van Pelt S.I., Ruivenkamp C.A., Kroes W.G., Cao J., Jager M.J., Luyten G.P.M., van der Velden P.A. (2015). Digital PCR validates 8q dosage as prognostic tool in uveal melanoma. PLoS ONE.

[22] Koopmans A.E., Verdijk R.M., Brouwer R.W.W., van den Bosch T.P.P., van den Berg M.M.P., Vaarwater J., Kockx C.E.M., Paridaens D., Naus N.C., Nellist M. (2014). Clinical significance of immunohistochemistry for detection of BAP1 mutations in uveal melanoma. Mod. Pathol..

[23] Zoutman W.H., Nell R., Versluis M., Van Steenderen D., Lalai R.N., Out-Luiting J.J., De Lange M.J., Vermeer M., Langerak A.W., Van Der Velden P.A. (2017). Accurate Quantification of T Cells by Measuring Loss of Germline T-Cell Receptor Loci with Generic Single Duplex Droplet Digital PCR Assays. J. Mol. Diagn..

[24] De Lange M.J., Nell R., Lalai R.N., Versluis M., Jordanova E.S., Luyten G.P., Jager M.J., Van Der Burg S.H., Zoutman W.H., van Hall T. (2018). Digital PCR-Based T-cell Quantification–Assisted Deconvolution of the Microenvironment Reveals that Activated Macrophages Drive Tumor Inflammation in Uveal Melanoma. Mol. Cancer Res..

[25] Luyten G.P., Naus N.C., Mooy C.M., Hagemeijer A., Kan-Mitchell J., Van Drunen E., Vuzevski V., De Jong P.T.V.M., Luider T.M. (1996). Establishment and characterization of primary and metastatic uveal melano-ma cell lines. Int. J. Cancer.

[26] Chen P.W., Murray T.G., Uno T., Salgaller M.L., Reddy R., Ksander B.R. (1997). Expression of MAGE genes in ocular melanoma during progression from primary to metastatic disease. Clin. Exp. Metastasis.

[27] Amirouchene-Angelozzi N., Némati F., Gentien D., Nicolas A., Dumont A., Carita G., Camonis J., Desjardins L., Cassoux N., Piperno-Neumann S. (2014). Establishment of novel cell lines recapitulating the genetic landscape of uveal melanoma and preclinical validation of mTOR as a therapeutic target. Mol. Oncol..

[28] Bronkhorst I.H.G., Jehs T.M.L., Dijkgraaf E.M., Luyten G.P.M., van der Velden P.A., van der Burg S.H., Jager M.J. (2014). Effect of Hypoxic Stress on Migration and Characteristics of Monocytes in Uveal Melanoma. JAMA Ophthalmol..

[29] Landreville S., Agapova O.A., Matatall K.A., Kneass Z.T., Onken M., Lee R.S., Bowcock A., Harbour J.W. (2012). Histone Deacetylase Inhibitors Induce Growth Arrest and Differentiation in Uveal Melanoma. Clin. Cancer Res..

[30] Sakamoto S., Potla R., Larner A.C. (2004). Histone Deacetylase Activity Is Required to Recruit RNA Polymerase II to the Promoters of Selected Interferon-stimulated Early Response Genes. J. Biol. Chem..

[31] Nusinzon I., Horvath C.M. (2003). Interferon-stimulated transcription and innate antiviral immunity require deacetylase activity and histone deacetylase 1. Proc. Natl. Acad. Sci. USA.

[32] Li M.-L., Su X.-M., Ren Y., Zhao X., Kong L.-F., Kang J. (2020). HDAC8 inhibitor attenuates airway responses to antigen stimulus through synchronously suppressing galectin-3 expression and reducing macrophage-2 polarization. Respir. Res..

[33] Balasubramanian S., Steggerda S., Sirisawad M., Schreeder M., Doiron L., Buggy J.J. (2008). The Histone Deacetylase-8 (HDAC8) Selective Inhibitor PCI-34051 Decreases Interleukin-1 Beta Secretion in Vitro and Reduces Inflammation in Vivo. Blood.

[34] Woan K.V., Sahakian E., Sotomayor E.M., Seto E., Villagra A. (2011). Modulation of antigen-presenting cells by HDAC inhibitors: Implications in autoimmunity and cancer. Immunol. Cell Biol..

[35] Charron C.E., Chou P.-C., Coutts D.J.C., Kumar V., To M., Akashi K., Pinhu L., Griffiths M., Adcock I., Barnes P.J. (2009). Hypoxia-inducible Factor 1α Induces Corticosteroid-insensitive Inflammation via Reduction of Histone Deacetylase-2 Transcription. J. Biol. Chem..

[36] Brouwer N.J., Wierenga A.P.A., Gezgin G., Marinkovic M., Luyten G.P.M., Kroes W.G.M., Versluis M., Van Der Velden P.A., Verdijk R.M., Jager M.J. (2019). Ischemia Is Related to Tumour Genetics in Uveal Melanoma. Cancers.

[37] Herlihy N., Dogrusöz M., van Essen T.H., Harbour J.W., van der Velden P.A., van Eggermond M.C.J.A., Haasnoot G.W., Elsen P.J.V.D., Jager M.J. (2015). Skewed Expression of the Genes Encoding Epigenetic Modifiers in High-Risk Uveal Melanoma. Investig. Ophthalmol. Vis. Sci..

[38] Leslie P.L., Chao Y.L., Tsai Y.-H., Ghosh S.K., Porrello A., Van Swearingen A., Harrison E.B., Cooley B.C., Parker J.S., Carey L.A. (2019). Histone deacetylase 11 inhibition promotes breast cancer metastasis from lymph nodes. Nat. Commun..

[39] Deng R., Zhang P., Liu W., Zeng X., Ma X., Shi L., Wang T., Yin Y., Chang W., Zhang P. (2018). HDAC is indispensable for IFN-γ-induced B7-H1 expression in gastric cancer. Clin. Epigenetics.

