# Supplementary material for: Expression of HDACs 1, 3 and 8 Is Upregulated in the Presence of Infiltrating Lymphocytes in Uveal Melanoma
Source: Cancers (Basel). 2021 Aug 18;13(16):4146. doi: 10.3390/cancers13164146 (PMC8393956; doi:10.3390/cancers13164146)
Supplement: Supplementary file 1 [file cancers-13-04146-s001.zip › cancers-1306888-supplementary.pdf]

Supplementary Table S1. Correlation between mRNA expression levels (determined by Illumina array) of different HDACs and expression of BAP1 in the TCGA cohort (n=80). R = two-tailed Spearman correlation coefficient. $P \leq 0.05$ is considered significant, and indicated in bold

	**BAP1**	
	**R**	***P***
**HDAC1**	-.201	.07
**HDAC2**	-.010	.93
**HDAC3**	-.268	**.02**
**HDAC4**	-.625	**<0.001**
**HDAC6**	.427	**<0.001**
**HDAC7**	-.022	.84
**HDAC8**	-.560	**<0.001**
**HDAC9**	-.069	-.069
**HDAC11**	.739	**<0.001**

Supplementary Table S2. Correlation between mRNA expression levels (determined by Illumina array) of different HDACs and T cell fraction (%) as determined by ddPCR (n=59). R = two-tailed Spearman correlation coefficient. $P \leq 0.05$ is considered significant, and indicated in bold

	**T cell fraction**	
	**R**	***P***
**HDAC1**	.399	**.002**
**HDAC2**	-.232	.08
**HDAC3**	.319	**.01**
**HDAC4**	.200	.13
**HDAC6**	-.314	**.02**
**HDAC7**	.183	.16
**HDAC8**	.357	**.01**
**HDAC9**	-.122	.36
**HDAC11**	-.363	**.005**

Supplementary Figure S1: Influence of adding IFNɤ on HLA-A and HLA-B mRNA expression on cultured UM cell lines.

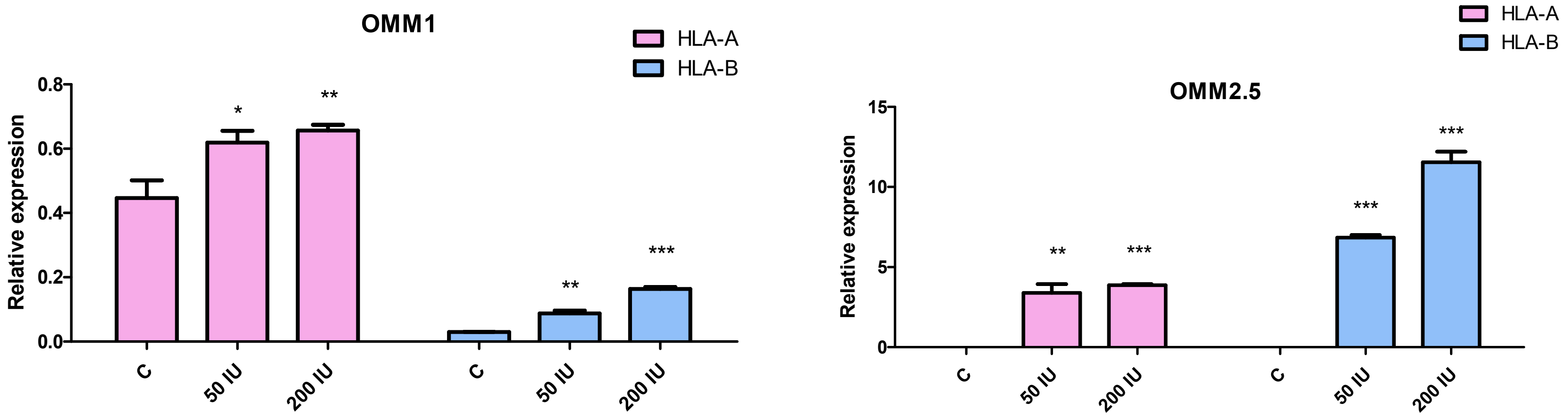

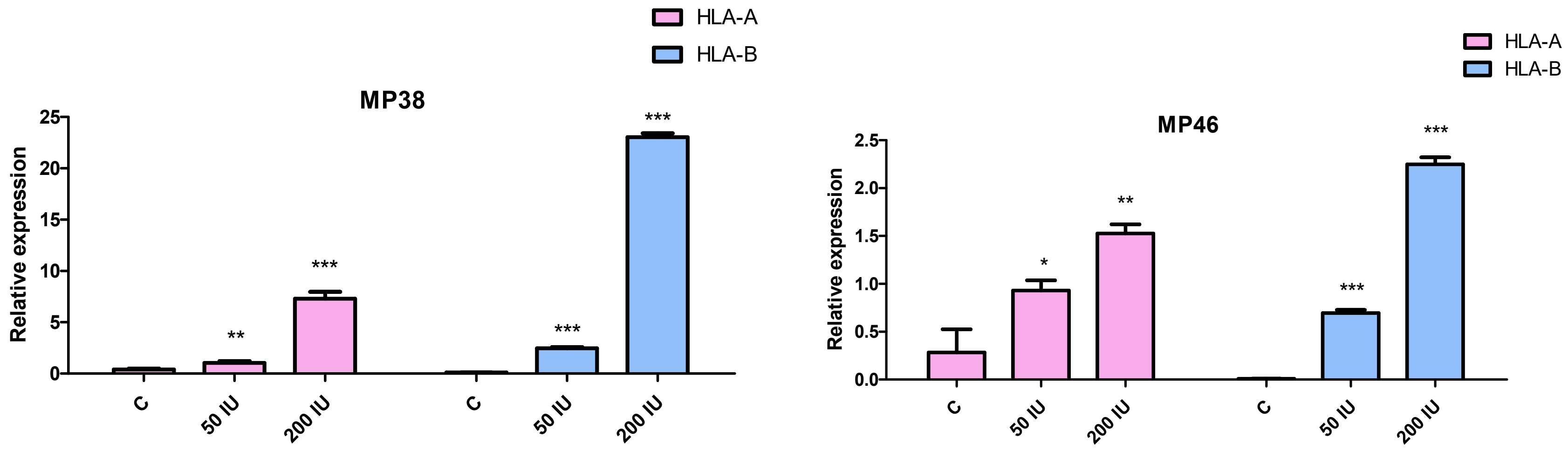

HLA-A
HLA-B
MP38
Relative expression
25
20
15
10
5
0
**
***
***
***
C
50 IU
200 IU
C
50 IU
200 IU
HLA-A
HLA-B
MP46
Relative expression
2.5
2.0
1.5
1.0
0.5
0.0
*
**
***
***
C
50 IU
200 IU
C
50 IU
200 IU
